# The Effective Prevention Program at 1 Hospital in China During the COVID-19 Epidemic

**DOI:** 10.1017/dmp.2021.36

**Published:** 2021-02-16

**Authors:** Huan Wang, Kaihang Sun, Lei Wang, Kai Zhang, Qilin Tang

**Affiliations:** 1 Outpatient Office, Tianjin Gong An Hospital, Tianjin, China; 2 Department of Acupuncture and Moxibustion, First Teaching Hospital of Tianjin, University of Traditional Chinese Medicine, Tianjin, China; 3 Department of General Surgery, Tianjin Gong An Hospital, Tianjin, China; 4 Department of Acupuncture and Moxibustion, Tianjin Gong An Hospital, Tianjin, China; 5 School of Basic Medical Sciences, Hebei University of Chinese Medicine, Hebei, Shijiazhuang, China

**Keywords:** China, COVID-19, hospital, prevention program

Since December 2019, the coronavirus disease (COVID-19) epidemic has spread rapidly from Wuhan,^1-3^ Hubei Province, China. Chinese hospitals have adopted strict measures, and this paper mainly reports on the protection measures of Tianjin Gong An Hospital (a general hospital in Tianjin, China). Tianjin is the largest port city in Northern China, with a permanent population of approximately 15.62 million. On January 21, 2020, Tianjin discovered the city’s first COVID-19 case. As of September 13, 2020, a total of 234 COVID-19 cases have been confirmed in Tianjin. On February 17, 2020, prevention strategies were implemented in Gong An Hospital, which is consistent with the preventive time of many communities. As of September 13, 2020, no doctors or patients have been infected in this hospital. The infection rate before and after the implementation of the prevention program in this hospital has always been maintained at 0.

**Figure 1.**
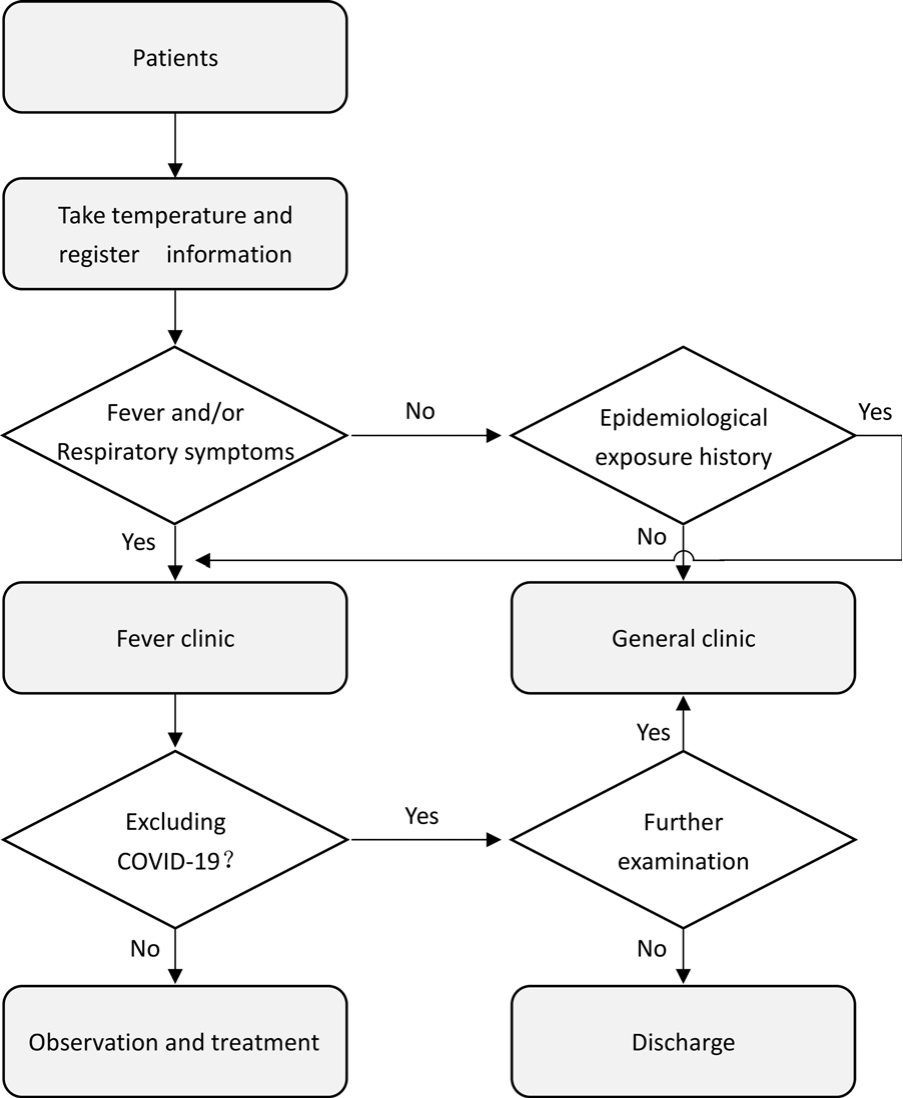
Visiting hospital flow chart.

First, the hospital opens a fever clinic to screen suspected cases. The fever clinic is separate from the general clinic buildings. Second, the hospital provides patients with a triage center. For example, it is important to ask patients whether they have traveled or lived in Hubei since the prior 2 weeks or whether they have been exposed to patients with fever and respiratory symptoms from Hubei in the 14 days before the onset of illness, and whether there was an epidemic of infectious diseases in the residential area.^4^ In addition, patients must be asked whether they have symptoms of respiratory infections, such as a cough. Furthermore, patients need to scan the QR code of the hospital through the social software on the mobile phone to enter the hospital. This operation can effectively identify the general information of patients and facilitate follow-up. For the elderly who do not use mobile phones, family members can operate on their behalf or register general information by handwriting. The visiting hospital flow chart is shown in Figure 1. Medical staff who register and take a patient’s temperature should wear protective clothing, protective glasses, masks, and gloves. The spread of COVID-19 through the eyes should not be ignored.^5^ Therefore, medical staff should be well protected. Doctors in all departments must wear surgical masks, and all doctors examining suspected cases should wear protective glasses. Ensuring daily cleaning and disinfection of the clinic is essential, including air and surface of objects.

China focuses on traditional public health epidemic response strategies. It seems that the strict measures taken by the Chinese health system may have prevented the further dissemination of the virus: severe acute respiratory syndrome coronavirus 2 (SARS-CoV-2). This study is limited to just 1 hospital in China. On the other hand, when it comes to the triage center, it is very useful to know whether all the people who come to the clinic are coming to receive emergency treatment, continued treatment, or preventive treatment, and whether the emergency treatment focuses on respiratory diseases or other emergencies. Furthermore, we may miss patients without respiratory symptoms. Unfortunately, we were not able to investigate the feelings of hospital staff about the epidemic prevention and control measures.
